# An Unbiased Feature Estimation Network for Few-Shot Fine-Grained Image Classification

**DOI:** 10.3390/s24237737

**Published:** 2024-12-03

**Authors:** Jiale Wang, Jin Lu, Junpo Yang, Meijia Wang, Weichuan Zhang

**Affiliations:** School of Electronic Information and Artificial Intelligence, Shaanxi University of Science and Technology, Xi’an 710000, China; 231612042@sust.edu.cn (J.W.); yangjunpo@foxmail.com (J.Y.); 4672@sust.edu.cn (M.W.)

**Keywords:** few-shot fine-grained image classification, data augmentation techniques, unbiased feature estimation network

## Abstract

Few-shot fine-grained image classification (FSFGIC) aims to classify subspecies with similar appearances under conditions of very limited data. In this paper, we observe an interesting phenomenon: different types of image data augmentation techniques have varying effects on the performance of FSFGIC methods. This indicates that there may be biases in the features extracted from the input images. The bias of the acquired feature may cause deviation in the calculation of similarity, which is particularly detrimental to FSFGIC tasks characterized by low inter-class variation and high intra-class variation, thus affecting the classification accuracy. To address the problems mentioned, we propose an unbiased feature estimation network. The designed network has the capability to significantly optimize the quality of the obtained feature representations and effectively reduce the feature bias from input images. Furthermore, our proposed architecture can be easily integrated into any contextual training mechanism. Extensive experiments on the FSFGIC tasks demonstrate the effectiveness of the proposed algorithm, showing a notable improvement in classification accuracy.

## 1. Introduction

Few-shot fine-grained image classification (FSFGIC) is a highly challenging task that aims to address the issue of overfitting in small sample settings [1], as well as the problem of low inter-class differences and high intra-class variations in fine-grained settings [2]. In recent years, with the continuous advancement of deep learning, many researchers have begun to focus on FSFGIC tasks [3,4,5]. Existing FSFGIC methods can be roughly classified into two groups [6]: meta-learning-based and metric-learning-based FSFGIC methods. Meta-learning-based methods [7,8] aim to enable a given model to learn quickly and obtain good generalization performance when adapting to new tasks in a scenario with limited data by optimizing model parameters or learning strategies. Metric-learning-based methods [9] use metric functions such as cosine distance or Euclidean distance to determine the category of samples based on the similarity between different samples.

**Figure 1:**
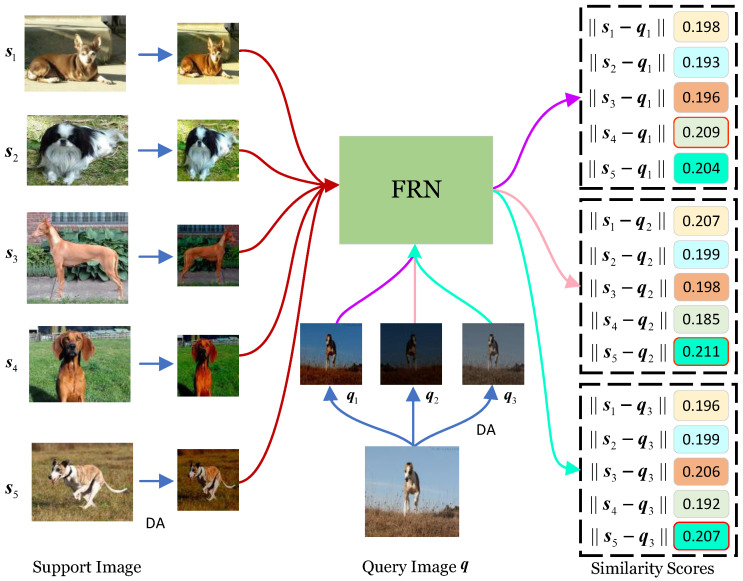
The impact of bias on obtaining feature representations. DA represents data augmentation operations. The numbers on the far right indicate the probability values of the predicted categories, with the red border highlighting the prediction with the highest probability.

In this work, the focus is on metric-learning-based FSFGIC. Generally, the metric-learning-based FSFGIC methods include two main components: a feature-embedding network and a similarity metric-learning network [10,11,12]. One of the main issues of the metric-learning methods is how to employ a convolution neural network (CNN) [13] with different similarity measures (e.g., nearest neighbor metric [14], hyperbolic distance [15], and cosine metric [16]) to learn an effective feature representation for each category. Although existing metric-learning-based FSFGIC methods have achieved a certain level of success, there are still some unresolved issues. Data augmentation (DA) techniques, such as random rotation [17], cropping [18], scaling [19], and color jittering [20], somewhat alleviate the class imbalance problem [21,22,23,24] associated with insufficient training data in FSFGIC. However, they also inevitably introduce quantization noise, leading to biased feature representations obtained by the convolutional neural network used as the feature-embedding module. Furthermore, the input images themselves are acquired through different sensors, which have different quantization noises [25]. Taking the feature map reconstruction networks (FRN) [26] as an example, as shown in Figure 1, the five support images belong to five different categories; the support image, $s_{5}$, and query image, q, belong to the same category, and DA operations with random cropping, random horizontal flipping, and color jittering are randomly performed on the six images. DA are carried out once on the five support images and DAs are carried out three times on the query image for obtaining three new query images (i.e., $q_{1}$, $q_{2}$, and $q_{3}$). Under the condition that all parameter settings remain unchanged, the five support images and the three query images after DA are sent into the trained FRN model, and their corresponding similarities are shown in Figure 1. Although objects of the same class have the same feature properties, it can be seen from the Euclidean distance in Figure 1 that only query images $q_{2}$ and $q_{3}$ and support image $s_{5}$ can be correctly classified into one category in these three classification instances. It can also be seen from Figure 1 that the similarities between a query image and different support images from different categories are very close. The reasons for this are as follows: (1) There is a bias in obtaining image feature information from a given CNN; (2) DA operation increases the quantization noise of images to a certain degree; (3) Fine-grained images have small inter-class and large intra-class variations. In addition, in the case of optical sensor data [27], the quality and characteristics of the sensor itself can also have an impact. For example, the resolution and sensitivity of an optical sensor may affect the sharpness and detail of the captured image, which in turn affects the accuracy of feature extraction and classification [28]. The lighting conditions in the environment used by the optical sensor can also cause changes in the image data, further complicating the classification task [29].

To address the mentioned problems, an unbiased feature estimation network (UFENet) is proposed. The contributions of this work can be summarized as follows:

- We are the first to discover and analyze that existing metric-learning-based FSFGIC algorithms are prone to bias in the feature information obtained from each input image due to image quantization noise. This is particularly relevant in the context of optical sensors, where data captured by optical sensors may be more susceptible to such quantization noise.
- An UFENet is proposed that has the capability to address the issue of bias of feature representations. In the field of optical sensor applications, it can improve the quality of feature extraction, reduce negative effects, and ensure accurate and reliable feature representation.
- The proposed UFENet has the capability to guide convolutional neural network (CNN)-based feature-embedding networks to achieve robust image feature representations for FSFGIC. Combining this with optical sensors enhances performance, helps in the adaptation to image features, and optimizes the extraction process to meet classification requirements.
- Experiments on four benchmark fine-grained datasets obtained through optical sensors (i.e., CUB-200-2011 [30], Stanford Dogs [31], Stanford Cars [32], and Aircraft [33]) demonstrate that the proposed UFENet significantly improves the performance of few-shot fine-grained image classification compared with the existing state-of-the-art methods.

The remainder of this article is organized as follows. In Section 2, we will discuss the literature closely related to our work. Section 3 presents the technical details of UFENet. In Section 4, we provide extensive experimental results and ablation studies. Finally, we conclude the article in Section 5.

## 2. Related Works

### 2.1. Metric-Learning-Based FSFGIC

The purpose of metric-learning-based methods is to learn a good task independent embedding for FSFGIC. The prototypical network (ProtoNet) [14] introduces the concept of class averaging, which involves using the average feature vector of all images in the support set belonging to the same class as the prototype for that class, and it then assigns the query image to the class whose prototype has the shortest Euclidean distance. The deep nearest neighbor neural network (DN4) [15] replaces image-level feature vectors with several local descriptors, implementing the cosine similarity measure between images and categories through summation. In the work of [20], a bi-similarity network (BSNet) was introduced, which utilizes the sum of two similarity metrics, namely the Euclidean distance and the cosine distance, to learn discriminative feature representations. In addition, hyperbolic distance [16] provides another option for distance measurement.

### 2.2. Attention Mechanism

Attention mechanisms were also widely employed in metric-learning-based FSFGIC algorithms, which have the capability to provide effective feature weighting strategies for metric learning, enabling a model to process complex data more effectively, thereby improving the classification performance and generalization ability of the model. In [34], a combination of multi-scale feature pyramids and multi-level attention pyramids enhances the internal representation of features and reduces the uncertainty caused by the background mediated by limited samples. In [35], visual self-attention mechanisms are used to infer local feature relationships, model spatial long-distance dependencies, estimate representative prototypes, and develop discriminative prototype-query pairs. HelixFormer [36] is a double-helix model based on Transformer, which addresses the FSFGIC task by learning the cross-image object semantic relationships in the local regions of images. Dual attention networks (DANs) [37] utilize spatial attention to capture the discriminative information of fine-grained images and employ channel attention to capture the global information of images. In the work of [38], a task difference maximization (TDM) module is introduced. Its goal is to locate regions. Task-specific channel weights are learned based on the support attention module (SAM) and the query attention module (QAM). The combination of the two is used to generate task-adaptive feature maps.

### 2.3. Feature Alignment

Feature alignment techniques have been introduced for learning feature embedding. A background suppression and foreground alignment network (BSFA) [39] was presented that aims to suppress the background content of images and align the foreground of support and query images. Ma et al. [40] proposed a cross-layer and cross-sample feature optimization network (C2-Net) that integrates feature maps from multiple network layers and improves the matching results between query features and support samples by adjusting the query features from both channel and spatial perspectives. Huang et al. [41] proposed a low-rank pairwise alignment bilinear network (LRPABN) that utilizes global feature alignment to learn a transformation matrix for minimizing the Euclidean distance between support and query image pairs.

### 2.4. Feature Reconstruction

Feature reconstruction techniques have also been widely applied in metric-learning-based FSFGIC tasks. Zhang et al. [42] proposed a method called DeepEMD based on the earth mover’s distance (EMD) [43] technique, which formulates feature reconstruction as an optimal transport problem for finding the best match between the query image and the support image. In order to take into account both the joint distribution of image features and lower computational overhead, Xie et al. [44] proposed a method based on deep Brownian distance covariance (DeepBDC), whose core idea is to learn image representations by measuring the difference between the joint characteristic function and the marginal product of the embedded features. In [26], FRN was introduced, which reconstructs query features directly from support features by ridge regression in closed form. Ref. [45] argued that existing reconstruction methods do not address the overfitting problem due to the scarcity of samples during training, so a self-reconstruction network (SRNet) based on FRN was proposed. In the work of [46], a local content enrichment cross-reconstruction network (LCCRN) was proposed, in which a local content enrichment module was designed to learn discriminative local feature representations and a cross-reconstruction module was introduced to combine these local features with the appearance details obtained from a separate embedding module to enhance the semantic understanding of the network.

All of the above metric-learning-based FSFGIC methods in our study utilize data analysis techniques such as random clipping, random level flipping, and color jitter to enhance the diversity of data samples, reduce overfitting, and improve classification accuracy. It is worth noting that the data processing of images inevitably generates quantization noise. Current FSFGIC methods do not address biases in the feature information obtained from each input image, which can lead to biased feature representations and misclassification. How to obtain the feature information from the image effectively and suppress the interference of quantization noise has been the key problem restricting the development of FSFGIC.

## 3. Proposed Method

### 3.1. Problem Statement

A typical FSFGIC setting contains a support set, S, and a query set, Q. Support set S contains C different image classes, and each class in C is composed of K labeled samples. Query set Q is composed of unlabeled samples. Set S and set Q have the same data-label space. The goal of FSFGIC is to train a model that has the ability to classify each query sample, q (q∈Q), into its corresponding class in C. Thus, the FSFGIC task is called a C-way K-shot task [47].

### 3.2. Motivation of the Proposed Method

This work is largely inspired by the experimental observations shown in Figure 1. Although, as described in [48], DA is ‘one of two main methods for combating overfitting’, performing data augmentation on each image inevitably leads to the generation of quantization noise, resulting in biased feature information extracted from each input image. This bias in feature representation significantly impacts the performance of FSFGIC. The main reasons are as follows: (1) The inter-class variation in fine-grained image categories is small, while the intra-class variation is large. (2) The training samples for FSFGIC are extremely limited. This presents a dilemma—on one hand, to ensure the diversity of data images and reduce overfitting, DA operations need to be applied to each image; on the other hand, we need to effectively address the bias in feature information caused by data augmentation.

It is well-known that Gaussian distribution is a widely used statistical model that can effectively capture data variability and central tendency, provided that the similarity distance, *d*, obtained from the distance calculated between the images of the support set and the query set, conforms to a Gaussian distribution with a mean of μ and a standard deviation of $\sigma^{2}$. This allows us to quantify and control the characteristics of the similarity distance in a probabilistic manner. The mean, μ, represents the central value or the typical value of the distance, while the standard deviation, $\sigma^{2}$, measures the spread or the variability of the distance around the mean.

The similarity distances obtained by calculating the distance between one support image and *n* query set images obtained after *n* DA operations can be expressed as $d_{1},d_{2},\ldots ,d_{n}$. By representing them as $d_{1}∼N\left(\mu_{1},\sigma_{1}^{2}\right)$, $d_{2}∼N\left(\mu_{2},\sigma_{2}^{2}\right)$, …, $d_{n}∼N\left(\mu_{n},\sigma_{n}^{2}\right)$, we are accounting for the fact that each of these similarity distances may have different statistical properties due to the variations introduced by the data augmentation process. Data augmentation is often used to increase the diversity and robustness of the data, but it can also introduce some noise and variability.

By summing $d_{1},d_{2},\ldots ,d_{n}$ and dividing by *n* to obtain a new similarity distance, *D*, we are aiming to reduce the impact of the noise introduced by data augmentation. The new similarity distance is $D=\frac{1}{n}\sum_{i=1}^{n}d_{i}$. Considering the properties of Gaussian distributions, when independent random variables that follow Gaussian distributions are combined in this way, the resulting distribution also has certain characteristics. If $d_{i}∼N\left(\mu_{i},\sigma_{i}^{2}\right)$ for i=1,2,…,n, and the $d_{i}$’s are assumed to be independent (which is a reasonable assumption in the context of data augmentation where each augmented sample is typically generated independently), then the sum $\sum_{i=1}^{n}d_{i}$ follows a Gaussian distribution with mean $\sum_{i=1}^{n}\mu_{i}$ and variance $\sum_{i=1}^{n}\sigma_{i}^{2}$. When we divide this sum by *n* to obtain *D*, the new distance descriptor, *D*, has mean $\mu_{D}=\frac{1}{n}\sum_{i=1}^{n}\mu_{i}$ and variance $\sigma_{D}^{2}=\frac{1}{n^{2}}\sum_{i=1}^{n}\sigma_{i}^{2}$.

The relationship between the new similarity distance, *D*, and the initial similarity distance, *d*, is as follows. The initial similarity distance, *d*, conforms to the Gaussian distribution $d∼N(\mu ,\sigma^{2})$. The new similarity distance, *D*, is obtained by averaging the *n* distance descriptors, $d_{i}$, after data augmentation. From the perspective of distribution characteristics, for the mean, if we assume that the data augmentation process does not change the mean in an ideal case (i.e., $\mu_{i}=\mu$), then the mean of *D*, $\mu_{D}=\mu$. However, in reality, $\mu_{i}$ may vary due to data augmentation, but $\mu_{D}$ still represents the comprehensive mean of the *n* $\mu_{i}$. Regarding the variance, the variance of *d* is $\sigma^{2}$, while the variance of *D*, $\sigma_{D}^{2}=\frac{1}{n^{2}}\sum_{i=1}^{n}\sigma_{i}^{2}$. Since n≥1, when the data augmentation does not overly change the variance of $d_{i}$ (i.e., $\sigma_{i}^{2}$ is relatively stable), $\sigma_{D}^{2}\leq \sigma^{2}$, indicating that *D* has a more concentrated distribution compared to *d*. Functionally, the initial similarity distance *d* is the basis, and *D* is designed to mitigate the noise impact of data augmentation. *D* inherits the basic information from *d* as it is composed of the augmented similarity distance related to *d*. The averaging process smoothes out the random fluctuations in the individual similarity distance. The noise in each augmented similarity distance is likely to be random and has both positive and negative components. When we sum them up and average them, these random noise components tend to cancel each other out to some extent. Secondly, by reducing the variance of the new similarity distance *D* (compared to the individual variances of the $d_{i}$’s), we make it more stable and less sensitive to the specific noise patterns in each augmented sample. This means that *D* is a more reliable representation that captures the essential information from the original distance descriptors while being less affected by the noise introduced by data augmentation.

Generally, a convolution embedding module demonstrates greater consistency in the feature attributes obtained from different DA operations on the same image than those obtained from different images under different DA operations. Based on the above analysis, we propose a bias-free feature acquisition mechanism for FSFGIC, performing DA operations randomly on both support images and query images (i.e., random cropping, random horizontal flipping, and color jittering). DA is applied once to the support image and *n* times to the query image, resulting in *n* augmented query images. The enhanced support and query images are then sent to a given convolution embedding module to obtain feature descriptors. Similarity is computed using the regularized feature descriptors of the augmented support images and the *n* regularized feature descriptors of the augmented query images. Finally, the mean of the sum of these *n* similarity values is taken as the similarity measure between the query image and the support image. In achieving the diversity of training samples through data augmentation, the proposed UFENet addresses the inherent bias problem caused by image quantization noise.

### 3.3. The Proposed UFENet

**Figure 2:**
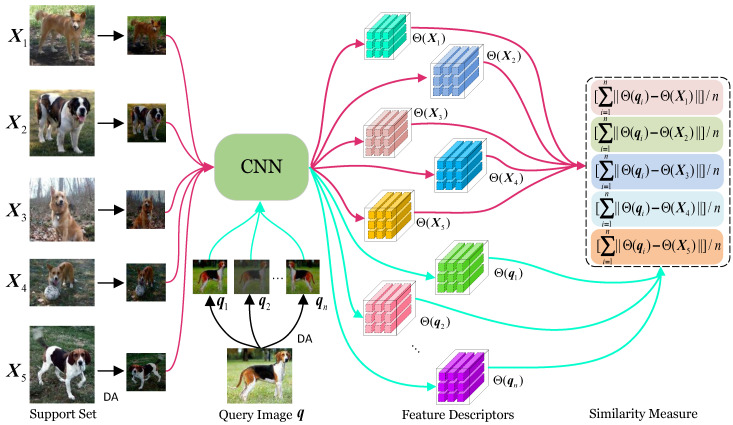
An overview of the proposed UFENet for a 5-way 1-shot FSFGIC task.

UFENet is designed to address the interference of quantization noise on feature descriptors while ensuring the diversity of training samples. Figure 2 illustrates the specific design architecture.

In this work, the most commonly used convolutional neural networks in the current FSFGIC methods, such as Conv-4 [26] and ResNet-12 [13], are used as the backbone network for feature extraction. For each query sample, q, and support sample, s, we apply DA operations consisting of random crop, random horizontal flip, and color jittering to the support sample and *n* iterations of the same operations to the query sample, resulting in the augmented query images ($q_{1}$, $q_{2}$, …, $q_{n}$). The augmented support and query images are fed into the given feature-embedding module to obtain feature descriptors, which are represented as follows: $\Theta \left(q_{1}\right)=\left[{\tilde{q}}_{1,1},{\tilde{q}}_{1,2},\ldots ,{\tilde{q}}_{1,m}\right]\in R^{d\times (h\times w)}$, $\Theta \left(q_{2}\right)=\left[{\tilde{q}}_{2,1},{\tilde{q}}_{2,2},\ldots ,{\tilde{q}}_{2,m}\right]\in R^{d\times (h\times w)}$, $\Theta \left(q_{n}\right)=\left[{\tilde{q}}_{n,1},{\tilde{q}}_{n,2},\ldots ,{\tilde{q}}_{n,m}\right]\in R^{d\times (h\times w)}$, and $\Theta \left(s\right)=\left[{\tilde{s}}_{1},{\tilde{s}}_{2},\ldots ,{\tilde{s}}_{m}\right]\in R^{d\times (h\times w)}$, where ${\tilde{s}}_{m}$ is the *m*-th feature descriptor with a length of *d*, *h* and *w* are the height and the width of the feature tensor map, *d* is the number of filters, R denotes real space, and *m* (m=h×w) is the total number of feature descriptors for training samples. In this way, the *z*-th feature descriptor ${\hat{q}}_{z}$(z=1,…,m) of query sample *q* is represented by ${\tilde{q}}_{1,z},{\tilde{q}}_{2,z},\ldots ,{\tilde{q}}_{n,z}$. Then, a standardization operation is performed on feature descriptors ${\tilde{q}}_{1,z},{\tilde{q}}_{2,z},\ldots ,{\tilde{q}}_{n,z}$ and ${\tilde{s}}_{z}$(z=1,…,m) as follows:

(1) $$\begin{array}{cc} {\ddot{q}}_{1,z} & =\frac{{\tilde{q}}_{1,z}-\Omega \left({\tilde{q}}_{1,z}\right)}{\sqrt{℘({\tilde{q}}_{1,z})}},{\ddot{q}}_{2,z}=\frac{{\tilde{q}}_{2,z}-\Omega \left({\tilde{q}}_{2,z}\right)}{\sqrt{℘({\tilde{q}}_{2,z})}},\ldots ,{\ddot{q}}_{n,z}=\frac{{\tilde{q}}_{n,z}-\Omega \left({\tilde{q}}_{n,z}\right)}{\sqrt{℘({\tilde{q}}_{n,z})}},\\ {\ddot{s}}_{z} & =\frac{{\tilde{s}}_{z}-\Omega \left({\tilde{s}}_{z}\right)}{\sqrt{℘({\tilde{s}}_{z})}},z=1,\ldots ,m, \end{array}$$

where Ω(·) and ℘(·) denote the mean operation and the variance operation, respectively. For each feature descriptor, ${\hat{q}}_{z}$ and ${\tilde{s}}_{z}$, they are sent to the feature reconstruction metric module [26], respectively. ${\hat{q}}_{z}$ is reconstructed by each ${\tilde{s}}_{z}$ via ridge regression as follows:

(2) $$\begin{array}{cc} {\bar{W}}_{1,z} & =\underset{W_{1,z}}{\mathrm{argmin}}\;||{\ddot{q}}_{1,z}-W_{1,z}\cdot {\ddot{s}}_{z}{\left|\right|}^{2}+\lambda ||W_{1,z}{\left|\right|}^{2},\\ {\bar{W}}_{2,z} & =\underset{W_{2,z}}{\mathrm{argmin}}\;||{\ddot{q}}_{2,z}-W_{2,z}\cdot {\ddot{s}}_{z}{\left|\right|}^{2}+\lambda ||W_{2,z}{\left|\right|}^{2},\\ \; & \vdots \\ {\bar{W}}_{n,z} & =\underset{W_{n,z}}{\mathrm{argmin}}\;||{\ddot{q}}_{n,z}-W_{n,z}\cdot {\ddot{s}}_{z}{\left|\right|}^{2}+\lambda ||W_{n,z}{\left|\right|}^{2}, \end{array}$$

where ||·|| is the Frobenius norm, λ is the regularization parameter, and ${\bar{W}}_{1,z}$, ${\bar{W}}_{2,z}$, *…*, ${\bar{W}}_{n,z}$ are the optimal weight matrices for reconstructing query images ${\bar{q}}_{1,z}$, ${\bar{q}}_{2,z}$, *…*, ${\bar{q}}_{n,z}$, as follows:

(3) $$\begin{array}{cc} {\bar{W}}_{1,z} & ={\tilde{q}}_{1,z}\cdot {\ddot{s}}_{z}^{T}{\left({\ddot{s}}_{z}\cdot {\ddot{s}}_{z}^{T}+\lambda I\right)}^{-1},\\ {\bar{q}}_{1,z} & ={\bar{W}}_{1,z}\cdot {\ddot{s}}_{z},\\ {\bar{W}}_{2,z} & ={\tilde{q}}_{2,z}\cdot {\ddot{s}}_{z}^{T}{\left({\ddot{s}}_{z}\cdot {\ddot{s}}_{z}^{T}+\lambda I\right)}^{-1},\\ {\bar{q}}_{2,z} & ={\bar{W}}_{2,z}\cdot {\ddot{s}}_{z},\\ \; & \vdots \\ {\bar{W}}_{n,z} & ={\tilde{q}}_{n,z}\cdot {\ddot{s}}_{z}^{T}{\left({\ddot{s}}_{z}\cdot {\ddot{s}}_{z}^{T}+\lambda I\right)}^{-1},\\ {\bar{q}}_{n,z} & ={\bar{W}}_{n,z}\cdot {\ddot{s}}_{z}. \end{array}$$

The Euclidean metric is utilized to compute the distance from query images ${\ddot{q}}_{1,z}$, ${\ddot{q}}_{2,z}$, *…*, ${\ddot{q}}_{n,z}$ to the reconstructed query images ${\bar{q}}_{1,z}$, ${\bar{q}}_{2,z}$, *…*, ${\bar{q}}_{n,z}$, as follows:

(4) $$\begin{array}{cc} M_{1,z} & =\frac{1}{r}\left|\right|{\ddot{q}}_{1,z}-{\bar{q}}_{1,z}{\left|\right|}^{2},\\ M_{2,z} & =\frac{1}{r}\left|\right|{\ddot{q}}_{2,z}-{\bar{q}}_{2,z}{\left|\right|}^{2},\\ \; & \vdots \\ M_{n,z} & =\frac{1}{r}\left|\right|{\ddot{q}}_{n,z}-{\bar{q}}_{n,z}{\left|\right|}^{2}. \end{array}$$

Furthermore, their corresponding similarity measure and the probability that the *j*-th query image belongs to the *c*-th class are as follows:

(5) $$\begin{array}{cc} M_{j}^{c} & =\frac{\sum_{i=1}^{n}M_{i,z}}{n},\\ {\hat{M}}_{j}^{c} & =\frac{e^{-\mu M_{j}^{c}}}{\sum_{c=1}^{C}e^{-\mu M_{j}^{c}}}, \end{array}$$

where *j* represents the *j*-th query image and μ is a learnable weight parameter. Finally, the stochastic gradient descent optimization [49] with a cross-entropy loss is used to train the whole network for performing FSFGIC tasks.

## 4. Experiments

### 4.1. Datasets

The proposed UFENet was evaluated on four fine-grained image datasets: CUB-200-2011 [30], Stanford Dogs [31], Stanford Cars [32], and Aircraft [33]. It is worth noting that these images were all obtained through optical sensors [27]. The CUB-200-2011 dataset contains 200 bird classes, with 11,788 samples. The Stanford Dogs dataset contains 120 dog classes, with 20,580 samples. The Stanford Cars dataset is composed of 196 car classes, with 16,185 samples. The aircraft dataset has 100 classes of aircraft, with a total of 10,000 images. We randomly divided the dataset into the training set, with 50 classes, the validation set, with 25 classes, and the test set, with 25 classes. For fair comparisons, we followed the data splits described in [2], which are shown in Table 1. The differences in the number of categories between the training, validation, and test sets were designed to ensure the model’s effectiveness and practicality. The training set needed to cover a broad range of categories to allow the model to learn diverse features and build a comprehensive knowledge base. The validation set was used to evaluate the model’s performance during training, typically with a simplified and representative set of categories, enabling timely adjustments to the model’s hyperparameters. The test set was used for the final evaluation of the model’s overall performance, and its category distribution had to balance comprehensiveness and relevance to real-world applications, avoiding being too complex or too simplistic in order to ensure the model’s suitability in practical scenarios.

### 4.2. Implementation Details

Experiments were conducted in the 5-way 1-shot and 5-way 5-shot FSFGIC settings on the four datasets mentioned above. All experiments in this work were conducted using the PyTorch 2.2.0 framework on 2 NVIDIA 3090 Ti GPUs through data parallelism. ResNet-12 and Conv-4 were selected as the backbones for obtaining feature representations using stochastic gradient descent [49] with a cross-entropy loss. The initial learning rate was 0.1, with weight decay set to $5\times 10^{-4}$. The learning rate was reduced to 0.01 after 400 iterations. For all experiments, this paper validated the average accuracy of 10,000 randomly generated tasks for obtaining the top-1 mean classification accuracy results under the standard 5-way 1-shot and 5-way 5-shot settings. Meanwhile, the 95% confidence intervals were obtained and reported.

### 4.3. Performance Comparison

In this part, the classification performance of the proposed UFENet is compared with fifteen state-of-the-art methods (i.e., Proto [14], DN4 [15], DSN [50], LRPABN [41], BSNet [20], FRN [26], Pacl [51], DAN [37], DeepEMD [42], FRN+TDM [38], DeepBDC [44], HelixFormer [36], LCCRN [46], BSFA [39], SRNet [45]). The experimental results on the CUB-200-2010, Stanford Dogs, Stanford Cars, and Aircraft datasets are summarized in Table 2. The results associated with the method marked by the † tag are derived from our implementation of the open-source code, conducted under the same experimental conditions.

**Figure 3:**
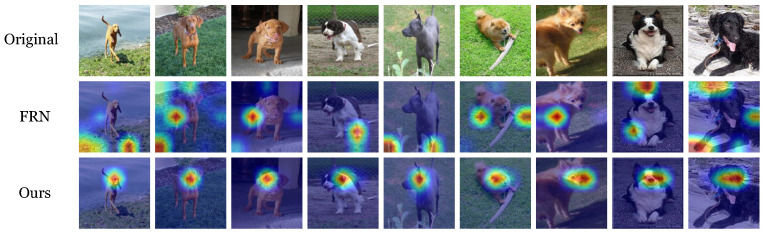
The heatmaps of nine images visualized by the FRN and the proposed UFENet.

Furthermore, taking nine images, as shown in Figure 3, as an example, the model attention region visualization technique based on the gradient-weighted class activation mapping (Grad-CAM) [52] on Conv-4 is utilized to illustrate the advantage of the proposed UFENet. In Grad-CAM, regions with higher energies represent more discriminative parts of the image. The attention maps of the six images of FRN and the proposed UFENet are shown in Figure 3, respectively. It can be observed from Figure 3 that, compared with FRN, the proposed UFENet has the capability to better focus on the classification targets themselves.

**Figure 4:**
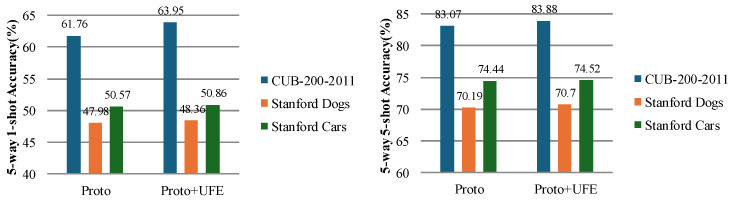
The proposed method is applied to the performance comparison of ProtoNet.

To further validate the effectiveness of the proposed algorithm, we applied the UFENet based on the Conv-4 backbone network to ProtoNet. Figure 4 illustrates the performance comparison of our algorithm under the 5-way 1-shot and 5-way 5-shot settings.

To evaluate the practical applicability of the proposed UFENet on edge devices, its FLOPs, parameter count, inference time, and FPS were tested, as shown in Table 3. The results indicate that, whether based on Conv-4 or ResNet-12, the network demonstrates excellent real-time performance, with a small parameter count, making it suitable for deployment on resource-constrained devices.

### 4.4. Ablation Studies

The performance of our designed UFENet architecture on FSFGIC tasks will be affected by the selection of parameter *n*. Additional experiments on FSFGIC were conducted to investigate the effect of the parameter *n* in Equation 5 on the performance. Based on the backbone of Conv-4 and ResNet-12, three different n(n∈3,4,5) were selected for performing 5-way 1-shot and 5-way 5-shot FSFGIC tasks on the CUB-200-2011 and Standford Dogs dataset. The results on the 5-way 1-shot and 5-way 5-shot tasks are shown in Table 4. It can be observed from Table 4 that when *n* equals 3, the proposed UFENet achieves the overall best classification performance. Therefore, DA operations on the query image with n=3 are recommended for our designed architecture.

## 5. Conclusions

In this paper, we first identified and analyzed a fundamental issue in existing metric-learning-based FSFGIC methods, especially in the context of optical sensors where data are often obtained via optical sensors. The issue is their inability to effectively address the bias in feature information obtained from each input image due to image quantization noise, which can lead to misclassification. Therefore, a simple yet effective UFENet is proposed to address image quantization noise and optimize feature extraction from optical sensor data. The architecture can be seamlessly integrated into any training framework, with validation on four benchmark datasets showcasing its superior performance compared to state-of-the-art methods, especially in optical sensor-based image scenarios. 

## Figures and Tables

**Table 1:** The class split of the four fine-grained datasets. $N_{\mathrm{train}}$, $N_{\mathrm{val}}$, and $N_{\mathrm{test}}$ are the numbers of classes in the auxiliary set, validation set, and test set, respectively.

Dataset	$N_{\mathrm{train}}$	$N_{\mathrm{val}}$	$N_{\mathrm{test}}$
CUB-200-2011	100	50	50
Stanford Dogs	70	20	30
Stanford Cars	130	17	49
Aircraft	100	50	50

**Table 2:** Comparison results of different methods on the CUB-200-2011, Stanford Dogs, Stanford Cars, and Aircraft datasets under two different backbones (methods labeled by ${\,}^{†}$ denote our implementations). The best performance is indicated in bold.

Backbone	Methods	CUB-200-2011		Stanford Dogs		Stanford Cars		Aircraft	
		**1-Shot**	**5-Shot**	**1-Shot**	**5-Shot**	**1-Shot**	**5-Shot**	**1-Shot**	**5-Shot**
Conv-4	Proto ${\,}^{†}$ [14]	61.76 ± 0.23	83.07 ± 0.15	47.98 ± 0.21	70.19 ± 0.16	50.57 ± 0.22	74.44 ± 0.17	-	-
	DN4 [15]	57.45 ± 0.89	84.41 ± 0.58	39.08 ± 0.76	69.81 ± 0.69	34.12 ± 0.68	87.47 ± 0.47	-	-
	DSN [50]	72.56 ± 0.92	84.62 ± 0.60	44.52 ± 0.82	59.42 ± 0.71	53.45 ± 0.86	65.19 ± 0.75	-	-
	LRPABN [41]	63.63 ± 0.77	76.06 ± 0.58	45.72 ± 0.75	60.94 ± 0.66	60.28 ± 0.76	73.29 ± 0.58	-	-
	BSNet [20]	62.84 ± 0.95	85.39 ± 0.56	43.42 ± 0.86	71.90 ± 0.68	40.89 ± 0.77	86.88 ± 0.50	56.51 ± 1.09	70.80 ± 0.81
	FRN ${\,}^{†}$ [26]	74.38 ± 0.21	88.69 ± 0.13	60.52 ± 0.22	79.29 ± 0.14	65.93 ± 0.22	87.35 ± 0.12	51.51 ± 0.21	71.99 ± 0.18
	Pacl [51]	74.07 ± 0.70	88.75 ± 0.38	59.76 ± 0.70	77.50 ± 0.48	**72.21 ± 0.68**	88.02 ± 0.36	-	-
	DAN [37]	72.89 ± 0.50	86.60 ± 0.31	59.81 ± 0.50	77.19 ± 0.35	70.21 ± 0.50	85.55 ± 0.31	-	-
	DeepEMD [42]	64.08 ± 0.50	80.55 ± 0.71	46.73 ± 0.49	65.74 ± 0.63	61.63 ± 0.27	72.95 ± 0.38	-	-
	ours	**75.67 ± 0.21**	**89.60 ± 0.12**	**61.08 ± 0.21**	**79.75 ± 0.14**	64.92 ± 0.22	**88.08 ± 0.12**	**59.49 ± 0.22**	**78.05 ± 0.17**
ResNet-12	Proto [14]	81.02 ± 0.20	91.93 ± 0.11	73.81 ± 0.21	87.39 ± 0.12	82.29 ± 0.20	93.11 ± 0.10	46.68 ± 0.81	71.27 ± 0.27
	FRN ${\,}^{†}$ [26]	83.94 ± 0.19	93.77 ± 0.10	77.89 ± 0.21	89.10 ± 0.12	89.43 ± 0.16	96.78 ± 0.07	70.68 ± 0.22	84.31 ± 0.14
	FRN+TDM [38]	83.26 ± 0.20	92.80 ± 0.11	75.98 ± 0.22	88.70 ± 0.13	86.91 ± 0.17	96.11 ± 0.07	70.61 ± 0.21	84.53 ± 0.13
	DeepBDC [44]	81.98 ± 0.44	92.24 ± 0.24	73.57 ± 0.46	86.61 ± 0.27	82.28 ± 0.42	93.51 ± 0.20	-	-
	DeepEMD [42]	75.59 ± 0.30	88.23 ± 0.18	70.38 ± 0.30	85.24 ± 0.18	80.62 ± 0.26	92.63 ± 0.13	69.86 ± 0.30	85.17 ± 0.28
	HelixFormer [36]	81.66 ± 0.30	91.83 ± 0.17	65.92 ± 0.49	80.65 ± 0.36	79.40 ± 0.43	92.26 ± 0.15	-	-
	LCCRN [46]	82.97 ± 0.19	93.63 ± 0.10	-	-	87.04 ± 0.17	96.19 ± 0.07	-	-
	BSFA [39]	82.27 ± 0.46	90.76 ± 0.26	69.58 ± 0.50	82.59 ± 0.33	88.93 ± 0.38	95.20 ± 0.20	-	-
	SRNet [45]	83.82 ± 0.18	93.45 ± 0.10	76.54 ± 0.21	88.52 ± 0.12	88.02 ± 0.16	96.23 ± 0.07	-	-
	ours	**84.90 ± 0.19**	**94.09 ± 0.09**	**78.61 ± 0.21**	**89.52 ± 0.11**	**89.82 ± 0.16**	**96.90 ± 0.06**	**86.80 ± 0.17**	**94.02 ± 0.08**

**Table 3:** A comprehensive evaluation of FLOPs, Params, Inference time, and FPS of UFENet: ’G’ stands for ’Giga’, ’M’ represents ’Mega’, and ’s’ stands for ’second’.

Backbone	FLOPs(G)	Para(M)	Inference Time(s)	FPS
Conv-4	0.099	0.113	0.003	305
ResNet-12	3.523	12.424	0.044	22

**Table 4:** The impact of the *n* DA operations of the proposed UFENet obtained on the CUB-200-2011 and Stanford Dogs datasets. The best performance is indicated in bold.

Backbone	*n*	CUB-200-2011		Stanford Dogs	
		**1-Shot**	**5-Shot**	**1-Shot**	**5-Shot**
Conv-4	2	75.36 ± 0.21	89.43 ± 0.12	60.65 ± 0.21	79.54 ± 0.14
	3	**75.67 ± 0.21**	**89.60 ± 0.12**	**61.08 ± 0.21**	**79.75 ± 0.14**
	4	75.33 ± 0.21	89.45 ± 0.12	60.52 ± 0.21	79.50 ± 0.14
ResNet-12	2	84.28 ± 0.18	93.19 ± 0.10	78.52 ± 0.21	89.41 ± 0.11
	3	**84.90 ± 0.19**	**94.09 ± 0.09**	**78.61 ± 0.21**	**89.52 ± 0.11**
	4	84.52 ± 0.19	93.79 ± 0.09	78.21 ± 0.21	89.30 ± 0.21

## Data Availability

Data are contained within the article.
